# Dysregulation of lnc-SNHG1 and miR-216b-5p correlate with chemoresistance and indicate poor prognosis of serous epithelial ovarian cancer

**DOI:** 10.1186/s13048-020-00750-4

**Published:** 2020-12-10

**Authors:** Mei Li Pei, Zong Xia Zhao, Ting Shuang

**Affiliations:** 1 Department of Gynecology and Obstetrics, The First Affiliated Hospital of Xi’an Jiao Tong University, Xi’an, 710061 China; 2 Department of Gynecology and Obstetrics, Xijing Hospital, Fourth Military Medical University, Xi’an, 710032 China; 3 Department of Gynecology and Obstetrics, The Second Affiliated Hospital of Xi’an Medical University, Xi’an, 710038 China

**Keywords:** Lnc-SNHG1, miR-216b-5p, ceRNA, Chemoresistance, Prognosis, Serous epithelial ovarian cancer

## Abstract

**Aim:**

This study aimed to explore whether the dysregulation of lnc-small nucleolar RNA host gene 1 (SNHG1) and miR-216b-5p correlated with chemoresistance and indicated poor prognosis of serous epithelial ovarian cancer (EOC).

**Methods and results:**

The expression of lnc-SNHG1 was upregulated, while miR-216b-5p showed low expression in patients with chemoresistant EOC compared with patients with chemosensitive EOC. The multivariate Cox regression analysis showed that the expression of miR-216b-5p and FIGO stage were independent prognostic factors for the overall survival (OS) of patients with serous EOC. Kaplan–Meier curves revealed a significant association of the increased expression level of lnc-SNHG1 with shorter OS and disease-free survival (DFS). Patients with a low expression level of miR-216b-5p also had shorter OS and DFS. The biological functions were tested using CCK-8 assay, colony formation assay, wound healing assay, and cell apoptosis. The knockdown of SNHG1 and the overexpression of miR-216b-5p stimulated paclitaxel sensitivity in A2780/Taxol cells through inhibiting cell growth and migration and promoting apoptosis. The inhibition of miR-216b-5p could rescue the effect of lnc-SNHG1 inhibition on the sensitivity of A2780/Taxol cells to paclitaxel. Luciferase reporter assay, RNA Binding Protein Immunoprecipitation Assay (RIP), and quantitative reverse transcription–polymerase chain reaction (qRT-PCR) indicated that lnc-SNHG1 acted as a sponge of miR-216b-5p in A2780/Taxol cells.

**Conclusions:**

This study showed that the overexpression of lnc-SNHG1 and decreased expression level of miR-216b-5p correlated with the chemoresistance of patients with serous EOC and indicated shorter OS and DFS. Lnc-SNHG1 functioned as a ceRNA with miR-216b-5p, which was critical in modulating the paclitaxel sensitivity of ovarian cancer cells.

**Supplementary Information:**

The online version contains supplementary material available at 10.1186/s13048-020-00750-4.

## Background

Over the past 30 years, the overall 5-year survival rate for patients with cancer has increased by 20% with early screening and therapy of cancer progression [1]. In contrast, the survival rate of patients with ovarian cancer has not changed much in recent decades; the 5-year survival rate is only 47% even in developed countries [2]. A total of 239,000 new cases of ovarian cancer occur annually worldwide (3.6% of all cancer cases), of which 152,000 cases die every year (4.3% of all cancer deaths). Ovarian cancer is also the seventh most common cancer among gynecological cancers and the first cause of death among women worldwide [3]. About 70% of patients have been diagnosed with stage III or stage IV ovarian cancer. The treatment using platinum along with paclitaxel after cytoreductive surgery followed by maintenance therapy is still the recommended standard treatment for ovarian cancer at home and abroad [4]. However, at least 70% of patients with ovarian cancer eventually develop chemotherapeutic resistance. Therefore, understanding the mechanism of ovarian cancer chemoresistance is essential.

Besides the application of high-throughput sequencing technology, the structure and function of noncoding RNA has gained attention. LncRNAs, as a class of noncoding RNA transcripts, are more than 200 nucleotides in length. LncRNAs do not have the potential to directly encode proteins due to the lack of open reading frames, but they can regulate gene expression in cis or trans through different mechanisms [5–7]. Studies showed that the dysregulation of lncRNAs was related to tumorigenesis, development, metastasis, and chemoresistance [8–10]. Further, lncRNAs have shown the potential as biomarkers in many malignant tumors [11, 12].

In 2011, Salmena et al. proposed a unique regulatory mechanism between lncRNAs and messenger RNAs, namely the competitive endogenous RNA (ceRNA) hypothesis. They assumed that lncRNA regulated the expression of mRNAs because it contained miRNA response element that could competitively bind to the same miRNAs [13]. That is to say, lncRNA could reduce the expression level of miRNA by sponge adsorption, thus inhibiting the negative regulation of miRNA on downstream target genes [14, 15]. The ceRNA regulatory model of competitive binding of lncRNA to miRNA has become a hotspot in many malignant tumors, including ovarian cancer. However, the study of the role of lncRNAs in the chemoresistance of ovarian cancer needs to be further explored. As a newly reported long noncoding RNA, lnc-small nucleolar RNA host gene 1 (SNHG1) was significantly overexpressed in lung cancer cell line than in normal lung epithelial cells; after silencing lnc-SNHG1 expression, the cell proliferation was inhibited [16]. Also, the expression of lnc-SNHG1 was significantly upregulated in liver cancer tissues and liver cancer cell line compared with normal liver tissue and cell line; the overexpression of lnc-SNHG1 promoted the proliferation, invasion, and migration of liver cancer cells through binding to miR-195 [17]. Whether lnc-SNHG1 is overexpressed in patients with chemoresistant ovarian cancer has not been reported.

Micro RNAs (miRNAs) play an important role in the regulation of gene expression related to cell growth cycle, cell proliferation, and apoptosis, and has been a hot topic in the study of chemoresistance in ovarian cancer. Li et al. reported that miR-142-5p enhanced the sensitivity of ovarian cancer cells to platinum by targeting and inhibiting the expression of anti-apoptotic genes [18]. Biamonte et al. claimed that microRNA let-7 g could be used as a tumor suppressor in epithelial ovarian cancer (EOC) and as a marker to predict the chemosensitivity of ovarian cancer [19]. Also, miR-34c and miR-383-5p increased the sensitivity of ovarian cancer cells to chemotherapy by inhibiting their proliferation [20, 21]. MiR-216b has been found to be involved in a variety of tumors except ovarian cancer in recent years. Wang [22] et al. found that the expression level of miR-216b was significantly lower in gastric adenocarcinoma than in normal tissues. However, whether the low expression of miR-216b-5p is related to the chemoresistance of ovarian cancer and its mechanism have not been reported.

This study found that the overexpression of lnc-SNHG1 and the decreased expression level of miR-216b-5p correlated with the chemoresistance of serous EOC and indicated less OS and shorter DFS of the patients. Lnc-SNHG1 acted as a sponge of rmiR-216b-5p in A2780/Taxol cells. The biological functions showed that the knockdown of SNHG1 and the overexpression of miR-216b-5p stimulated paclitaxel sensitivity in A2780/Taxol cells. In contrast, the inhibition of miR-216b-5p could rescue the effect of lnc-SNHG1 inhibition on the sensitivity of A2780/Taxol cells to paclitaxel.

## Results

### Expression levels of lnc-SNHG1 and miR-216b-5p and their clinical significance in patients with serous EOC

**Fig. 1:**
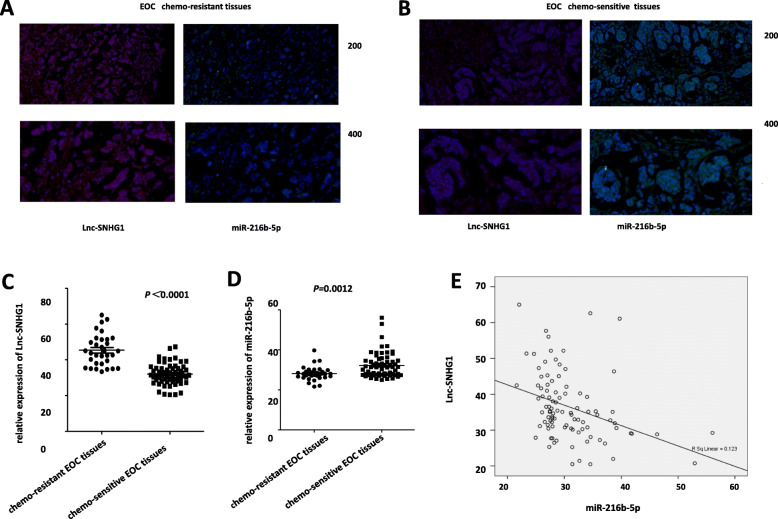
Expression and cellular localization of lnc-SNHG1 and miR-216b-5p were tested by fluorescence in situ hybridization (FISH) analysis. **a** Expression of lnc-SNHG1 and miR-216b-5p in chemoresistant tissues. **b** Expression of lnc-SNHG1 and miR-216b-5p in chemosensitive tissues. **c**-**d** Lnc-SNHG1 was significantly upregulated in chemoresistant serous EOC tissues, while miR-216b-5p showed low expression in chemoresistant serous EOC specimens compared with chemosensitive tissues. **e** Spearman’s correlation analysis was applied and showed correlations between lnc-SNHG1 and miR-216b-5p in patients with serous EOC. The correlation coefficient “*r*” was calculated

**Fig. 2:**
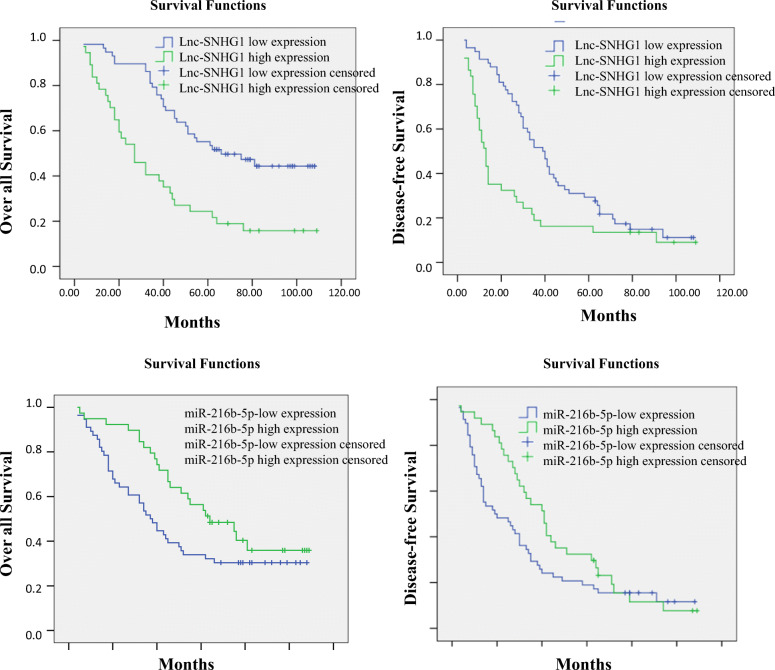
Kaplan–Meier survival analysis was used for the correlation of lnc-SNHG1 and miR-216b-5p expression with the OS and DFS of patients with serous EOC. **a** Patients with high lnc-SNHG1 expression showed significantly shorter OS (*P* = 0.019, survival interval: 59.045 ± 4.373 vs 41.714 ± 4.3 months); and shorter DFS (*P* = 0.019, survival interval: 59.045 ± 4.373 vs 41.714 ± 4.3 months) compared with those with low lnc-SNHG1 expression. **b** Patients with low miR-216b-5p expression showed significantly shorter OS (*P* = 0.019, survival interval: 59.045 ± 4.373 vs 41.714 ± 4.3 months) and shorter DFS (*P* = 0.019, survival interval: 59.045 ± 4.373 vs 41.714 ± 4.3 months) compared with those with high miR-216b-5p expression

The FISH analysis showed that lnc-SNHG1 expression was upregulated in patients with chemoresistant serous EOC compared with those with chemosensitive EOC, and it was mainly localized in the cytoplasm. However, the expression level of miR-216b-5p was low in chemoresistant tissues (Fig. 1a-b). Likewise, data showed that the expression level of lnc-SNHG1 was significantly higher in the chemoresistant group compared with the chemosensitive group (86.49% vs 13.51%, *P* = 0.0001) (Fig. 2c), whereas the expression level of miR-216b-5p was remarkably lower in the chemoresistant group compared with the chemosensitive group (10.81% vs 89.19%%, *P* = 0.0001) (Fig. 1d).

The expression level of lnc-SNHG1 was classified as low (*n* = 58) or high (*n* = 37) according to the median value of lnc-SNHG1 expression to assess whether the dysregulation of lnc-SNHG1 or miR-216b-5p was associated with the clinicopathological features of patients with serous EOC. Also, patients with EOC were categorized into miR-216b-5p low-expression group (*n* = 58) and miR-216b-5p high-expression group (*n* = 37) based on the median value of miR-216b-5p. The lnc-SNHG1 high expression showed a significant correlation with tumor stage, histological grade, nodal status, metastasis, and chemoresistance except tumor size (Table 1). However, no significant association was found between miR-216b-5p expression and parameters including tumor stage, histological grade, nodal status, and metastasis, but a significant correlation was observed with chemosensitivity (*P* = 0.0001, Table 2).

**Table 1:** Expression of lnc-SNHG1 in serous epithelial ovarian cancer tissues

		lnc-SNHG1					
	Total cases	High expression (***n*** = 37)		Low expression (***n*** = 58)		***χ***^**2**^	***P***
**Age (year)**	*n*	*n*	%	*n*	%	0.074	0.786
**Postmenopausal (< 50)**	42	17	40.48	25	59.52		
**Premenopausal (≧50)**	53	20	37.74	33	62.26		
**Tumor stage (FIGO)**						8.435	0.015
**I-II**	21	4	19.05	17	80.95		
**III**	45	16	35.56	29	64.44		
**IV**	29	17	58.62	12	41.38		
**Histological grade (*n*** **= 88)**						6.529	0.011
**H**	72	34	47.22	38	52.78		
**L**	16	2	12.50	14	87.50		
**Nodal status (*n*)**						13.323	0.0001
**Negative**	60	15	25.00	45	75.00		
**Positive**	35	22	62.86	13	37.14		
**Tumor diameter (cm)**						0.343	0.842
**< 10 cm**	30	12	40.00	18	60.00		
**10–20 cm**	58	23	39.66	35	60.34		
**> 20 cm**	7	2	28.57	5	71.43		
**Chemotherapy**						31.148	0.0001
**Chemoresistance**	32	25	78.13	7	21.87		
**Chemosensitivity**	63	12	19.05	51	80.95		
**Metastasis**						25.499	0.0001
**Y**	19	17	89.47	2	10.53		
***N***	76	20	26.32	56	73.68

**Table 2:** Expression of miR-216b-5p in serous epithelial ovarian cancer tissues

		miR-216b-5p					
	Total cases	High expression (***n*** = 37)		Low expression (***n*** = 58)		***χ***^**2**^	***P***
	n	n	%	n	%		
**Age (year)**						0.074	0.786
**Postmenopausal(< 50)**	42	17	40.48	25	59.52		
**Premenopausal (≧50)**	53	20	37.74	33	62.26		
**Tumor stage (FIGO)**						2.708	0.258
**I-II**	21	8	38.10	13	61.90		
**III**	45	21	46.67	24	53.33		
**IV**	29	8	27.59	21	72.41		
**Histological grade (*n*** **= 88)**						2.216	0.137
**H**	72	26	36.11	46	63.89		
**L**	16	9	56.25	7	43.75		
**Nodal status (*n*)**						2.509	0.113
**Negative**	60	27	45.00	33	55.00		
**Positive**	35	10	28.57	25	71.43		
**Tumor diameter (cm)**						1.065	0.587
**< 10 cm**	30	11	36.67	19	63.33		
**10–20 cm**	58	22	37.93	36	62.07		
**> 20 cm**	7	4	57.12%	3	42.88		
**Chemotherapy**						14.194	0.0001
**Chemoresistance**	32	4	12.50	28	87.50		
**Chemosensitivity**	63	33	52.38	30	47.62		
**Metastasis**						0.1	0.752
**Y**	19	8	42.11	11	57.89		
**N**	76	29	38.16	47	61.84		

Further, Spearman’s correlation analysis revealed a significantly negative correlation between lnc-SNHG1 and miR-216b-5p (*r* = − 0.424, *P* = 0.0001) (Fig. 1e).

### Risk factors related to chemoresistance of patients with serous EOC

The univariate analysis showed that the dysregulation of lnc-SNHG1 (*P* < 0.0001, Table 1) and miR-216b-5p (*P* = 0.0001, Table 2) level was indicated to be an independent related risk factor for the chemoresistance of patients with serous EOC. Besides, the multivariate logistic regression analysis was also used in this study. The clinical features including tumor stage, histological grade, nodal status, metastasis, tumor size, and expression levels of lnc-SNHG1 and miR-216b-5p were included in the model. The forward stepwise selection method was used to analyze variables. The results showed that the dysregulation of lnc-SNHG1 (*P* < 0.0001) and miR-216b-5p (*P =* 0.033) and the FIGO stage (*P* = 0.01) were factors associated with the chemoresistance of patients with serous EOC (Table 3).

**Table 3:** Multivariable analysis of factors associated with the chemoresistance of serous EOC

	***B***	S.E.	Sig.	Exp(B)	95% CI for EXP(B)	
					Lower	Upper
**Lnc-SNHG1**	2.159	0.583	0.0001	8.659	2.761	27.155
**Tumor stage (FIGO)**	1.049	0.408	0.01	2.856	1.283	6.355
**miR-216b-5p**	1.482	0.693	0.033	4.4	1.131	17.122

### Multivariate cox regression model result for OS and DFS

Multivariate Cox regression analysis was used to assess the prognostic factors for the OS and DFS of patients with serous EOC. The clinical features including tumor stage, histological grade, nodal status, metastasis, tumor size, and expression levels of lnc-SNHG1 and miR-216b-5p were included as variables in the analysis. The expression of miR-216b-5p (*P* = 0.012, RR 2.137, 95% CI 1.109–5.339) and FIGO stage (*P* = 0.001, RR 3.537, 95% CI 1.72–7.276) were independent prognostic factors for the OS of patients with serous EOC (Table 4). However, the FIGO stage (*P* = 0.003, RR 2.237, 95% CI 1.323–3.783) was the independent prognostic factor for the DFS of patients with serous EOC (Table 5).

**Table 4:** Multivariable Cox regression analyses for overall survival

	***B***	SE	Sig.	Exp(B)	95% CI for Exp(B)	
					Lower	Upper
**Age (year)**	−0.005	0.28	0.987	0.995	0.575	1.723
**Lnc-SNHG1**	0.422	0.317	0.183	1.525	0.819	2.842
**Nodal status**	0.395	0.401	0.325	1.484	0.676	3.256
**Metastasis**	− 0.471	0.478	0.325	0.624	0.244	1.594
**Tumor stage (FIGO)**	1.263	0.368	0.001	3.537	1.72	7.276
**miR-216b-5p**	0.759	0.304	0.012	2.137	1.178	3.875

**Table 5:** Multivariable Cox regression analyses for disease-free survival

	***B***	SE	Sig.	Exp(B)	95% CI for Exp(B)	
					Lower	Upper
**Age (year)**	0.08	0.25	0.749	1.083	0.664	1.768
**Lnc-SNHG1**	0.389	0.269	0.148	1.475	0.871	2.498
**Nodal status**	0.188	0.337	0.577	1.207	0.623	2.339
**Metastasis**	−0.053	0.401	0.895	0.949	0.432	2.082
**Tumor stage (FIGO)**	0.805	0.268	0.003	2.237	1.323	3.783
**miR-216b-5p**	0.465	0.253	0.066	1.591	0.97	2.611

### Comparison of OS and DFS using Kaplan–Meier analysis

Patients were followed up for 5–9 years in the present study. Kaplan–Meier analysis indicated that patients with a high expression level of lnc-SNHG1 had significantly less OS compared with patients with a low expression level of lnc-SNHG1 (survival interval: 40.068 ± 5.678 vs 71.329 ± 4.730, *P* < 0.0001). Further, the DFS also significantly decreased for the patients with a high expression level of lnc-SNHG1 (survival interval: 27.081 ± 5.345 vs 46.238 ± 4.022, *P* = 0.003). However, patients with a low expression level of miR-216b-5p had shorter OS (survival interval: 51.875 ± 5.267 vs 69.373 ± 5.601, *P* = 0.010) and DFS (survival interval: 33.978 ± 4.413 vs 45.949 ± 4.823, *P* = 0.007). Together, Kaplan–Meier analysis showed that lnc-SNHG1 overexpression was associated with a significant decrease in the mortality rate and DFS, while a low expression level of miR-216b-5p indicated poor OS and DFS (Fig. 2).

### Lnc-SNHG1 acted as a sponge for miR-216b-5p in ovarian cancer cells

**Fig. 3:**
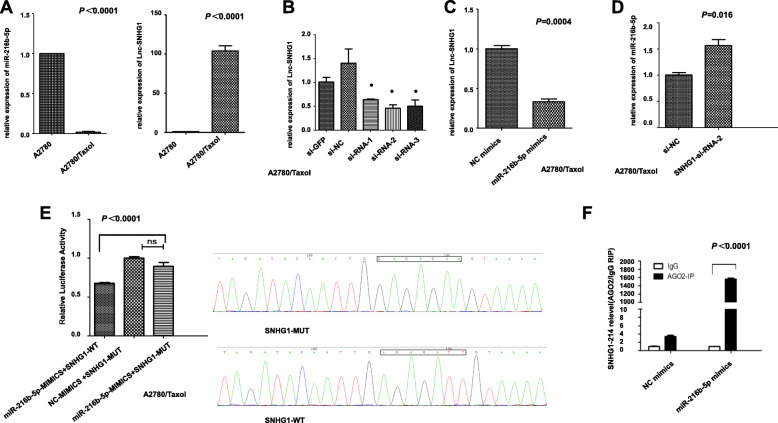
Lnc-SNHG1 acted as a sponge for miR-216b-5p in A2780/Taxol cells. **a** Expression level of lnc-SNHG1 and miR-216b-5p in A2780/Taxol and its parental A2780 cells. **b** A2780/Taxol cells were transfected with SNHG1-specific siRNAs; SNHG1- siRNA-2 was used for further investigation. **c, d** qRT-PCR analysis showed that miR-216b-5p was negatively regulated by SNHG1 while SNHG1 was negatively regulated by miR-216b-5p. **e**-**f** Luciferase reporter assay indicated that miR-216b-5p reduced the luciferase activity of SNHG1-WT rather than SNHG1-mut. L A2780/Taxol cells were transfected with MS2-tagged SNHG1-WT and MS2-tagged SNHG1-mut, and then were assayed by RIP

The ceRNA regulatory model of competitive binding of lncRNA to miRNA has become a hotspot in many malignant tumors. This study further explored whether lnc-SNHG1 bound to miR-216b-5p to affect the paclitaxel sensitivity of ovarian cancer cells. By applying starBase v2.0 [23], it was observed that lnc-SNHG1 could directly target miR-216b-5p (Fig. S1). We further found lnc-SNHG1 showed significantly up-regulation in A2780/Taxol cells than its parental A2780 cells, while the expression of miR-216b-5p showed the opposite trend (Fig. 3a). A2780/Taxol cells were transfected with siRNA of SNHG1 (si-SNHG1–1, si-SNHG1–2, and si-SNHG1–3) to explore the function of lnc- SNHG1 in A2780/Taxol cells, qRT-PCR was used to test the efficiency of transfection. Three SNHG1-specific siRNAs targeting different regions of SNHG1 were designed and introduced into A2780/Taxol cells. As shown in Fig. 3b, siRNA-1 and siRNA-2 effectively silenced the expression of lnc-SNHG1, therefore, siRNA-2 was used for further investigation. The expression level of lnc-SNHG1 was decreased to confirm the potential interaction between lnc-SNHG1 and miR-216b-5p, revealing correspondingly elevated expression level of miR-216b-5p (Fig. 3c). Further, miR-216b-5p expression was negatively regulated by transfecting A2780/Taxol cells with SNHG1-siRNA-2(Fig. 3d). Then, luciferase reporter assays were performed with A2780/Taxol cells. The results showed that the co-transfection of miR-216b-5p-mimics + SNHG1-WT showed significantly decreased fluorescence intensity compared with the co-transfection of miR-216b-5p -mimics and SNHG1-MUT, while miR-216b-5p-mimics+SNHG1-MUT-transfected A2780/Taxol cells showed no significant change compared with NC-mimics + SNHG1-MUT, indicating the interaction between miR-216b-5p -mimics and SNHG1-WT (Fig. 3e). RIP analysis was performed to further validate the direct interaction between miR-216b-5p and lnc-SNHG1. The results of RIP-qPCR showed that MS2-tagged wild-type SNHG1 (SNHG1-WT-MS2) was significantly enriched for miR-216b-5p in A2780/Taxol cells compared with the miRNA mimic NC (Fig. 3f). Taken together, these results indicated that lnc-SNHG1 could acted as a sponge for miR-216b-5p in A2780/Taxol cells.

### Knockdown of SNHG1 and overexpression of miR-216b-5p stimulated paclitaxel sensitivity in A2780/Taxol cells

**Fig. 4:**
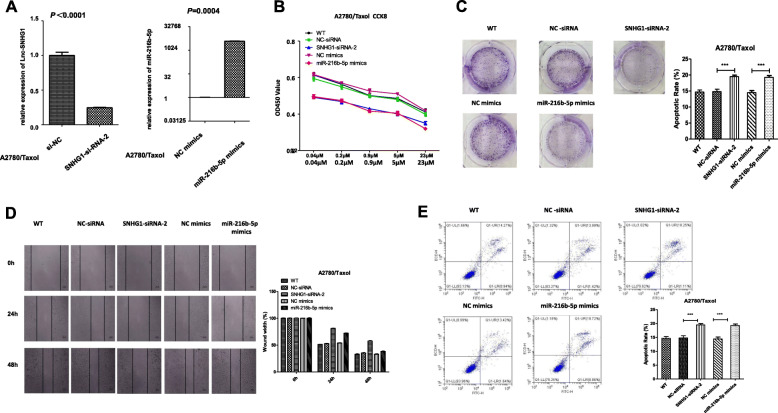
Knockdown of lnc-SNHG1 and overexpression of miR-216b-5p stimulated paclitaxel sensitivity in A2780/Taxol cells through inhibiting cell growth and migration and promoting apoptosis. **a** qRT-PCR analysis of lnc-SNHG1 and miR-216b-5p expression in A2780/Taxol cell lines after transfecion. **b** and **c** Proliferation capacity was determined after the downregulation of SNHG1 and the overexpression of miR-216b-5p in A2780/Taxol cells using CCK-8 assay and colony formation assay. **d** Migration ability was determined using the wound healing assay after transfection. **e** Flow cytometry analysis of apoptosis was performed. GAPDH was used as the control. ^***^P < 0.001

To explore the function of lnc- SNHG1 and miR-216b-5p in paclitaxel sensitivity of ovarian cancer cells. A2780/Taxol cells were transfected with si-SNHG1–2(siRNA NC). Also, the expression of level miR-216b-5p was elevated by transfecting miR-216b-5p (miRNA mimic NC), and qRT-PCR was used to test the efficiency of transfection (Fig. 4a). The functional assays were applied after the transfection and treated with paclitaxel for 24 h. The CCK-8 assays showed that the proliferation was inhibited significantly of A2780/Taxol cells transfected with miR-216b-5p mimic or siRNA -SNHG1–2 than that of A2780/Taxol cells transfected with miRNA mimic NC or siRNA NC (Fig. 4b). The colony formation assay confirmed that the number of colonies was reduced by SNHG1 deficiency and elevated expression of miR-miR-216b-5p (Fig. 4c). The wound healing assay showed that cell migration decreased after SNHG1 knockdown and overexpression of miR-216b-5p(Fig. 4d). Finally, the cell apoptosis assay showed that the apoptosis was accelerated by SNHG1silencing and miR-216b-5p overexpression (Fig. 4e). These results showed that the decreased expression level of lnc-SNHG1 and overexpression of miR-216b-5p could stimulated paclitaxel sensitivity in A2780/Taxol cells.

### Inhibition of miR-216b-5p could rescue the effect of lnc-SNHG1 inhibition on the sensitivity of A2780/Taxol cells to paclitaxel

**Fig. 5:**
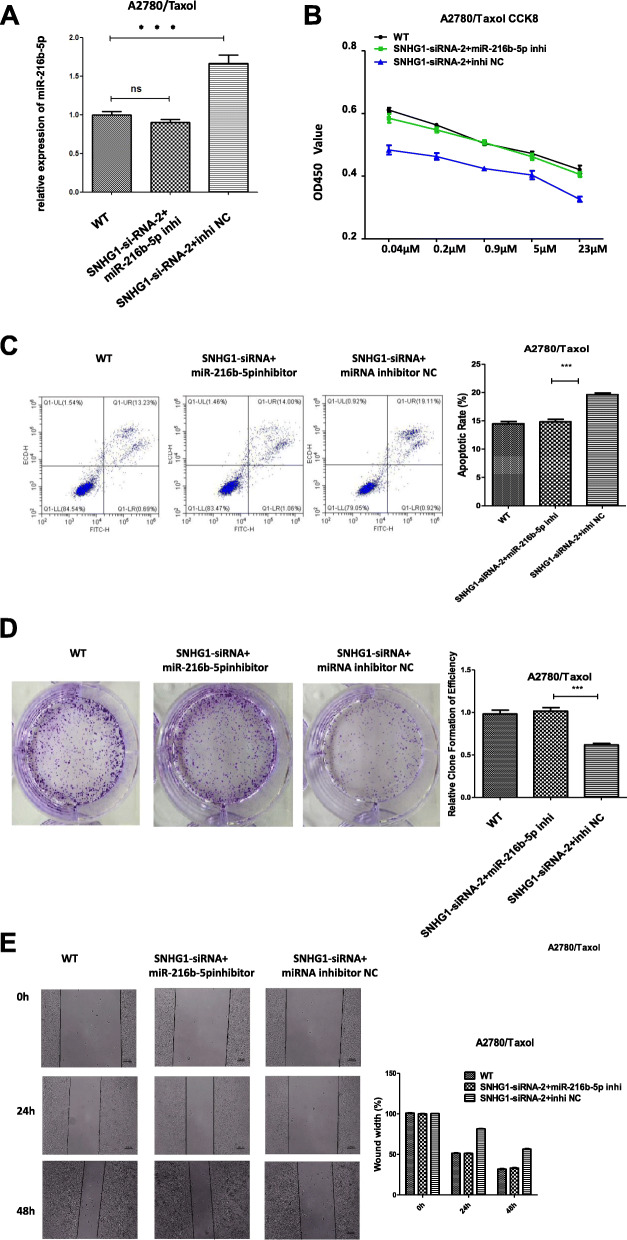
Inhibition of miR-216b-5p could rescue the effect of lnc-SNHG1 inhibition on the sensitivity of A2780/Taxol cells to paclitaxel. **a** qRT-PCR analysis of miR-216b-5p expression in A2780/Taxol cell lines after transfecion. **b**-**e** Biological functions of A2780/Taxol cells co-transfected with si-SNHG1 and miR-216b-5p inhibitor (si-SNHG1 + miR inhibitor NC group; WT used as the control group) were measured. **b** The CCK-8 test results showed that A2780/Taxol cells co-transfected with si-SNHG1 and miR-216b-5p inhibitor had significantly higher cell proliferation compared with the si-SNHG1 + miR inhibitor NC group. **c**-**e** Co-transfection with si-SNHG1 and miR-216b-5p inhibitor remarkably led to a decrease in apoptosis, but increased cell migration and number of colonies of A2780/Taxol cells. ^***^P < 0.001

A “rescue” experiment was performed to investigate whether miR-216b-5p could mediate the effects of lnc-SNHG1 inhibition, which stimulated paclitaxel sensitivity. A2780/Taxol cells were co-transfected with si-SNHG1 and miR-216b-5p inhibitor (si-SNHG1–2+ miR inhibitor NC group; WT as the control group). CCK-8 assay results showed that A2780/Taxol cells co-transfected with si-SNHG1 and miR-216b-5p inhibitor showed significantly higher cell proliferation compared with the si-SNHG1 + miR inhibitor NC (Fig. 5b). Co-transfection with si-SNHG1 and miR-216b-5p inhibitor remarkably led to a decrease in apoptosis (Fig. 5c), but increased cell migration (Fig. 5d) and number of colonies of A2780/Taxol cells (Fig. 5e).

## Discussion

Patients routinely accept chemotherapy using platinum along with paclitaxel after cytoreductive surgery followed by maintenance therapy for treating ovarian cancer. However, chemoresistance is still the main obstacle in successfully treating ovarian cancer and indicates poor prognosis of patients with ovarian cancer. Recent studies showed that lncRNAs were involved in the chemoresistance of cancers [24]. The dysregulation of lncRNAs in the chemoresistance of ovarian cancer has also gained much attention. Before 2015, it was reported that high expression level of HOTAIR could induce platinum resistance in ovarian cancer cells via DNA methylation [25]. In addition, the overexpression of lncRNA-FER1L4 could increase the sensitivity of ovarian cancer cells to paclitaxel by inhibiting the MAPK signaling pathway [26]. Also, lncRNA-KB-1471A8.2 was reported to be downregulated in ovarian cancer tissues and chemoresistant ovarian cancer cells and acted as tumor suppressor gene by inhibiting the expression of CDK4 [27].

As a newly discovered lncRNA, lnc-SNHG1 is localized at 11q12.3, and has been shown to be upregulated in many malignant tumors. You et al. reported that lnc-SNHG1 was significantly highly expressed in lung cancer cell lines than in normal lung epithelial cells; the cell proliferation was inhibited by silencing lnc-SNHG1 expression, [16]. Similarly, Wang et al. reported that lnc-SNHG1 was significantly upregulated in glioma tissues and associated with the poor OS of patients with glioma [28]. Interestingly, Zhang et al. claimed that lnc-SNHG1 was upregulated in HCC tissues than in adjacent liver tissues. They found that the high expression level of lnc-SNHG1 was closely related to large tumor size, poor differentiation, and aggressive stage, suggesting the poor prognosis of patients with HCC [29]. In 2019, a meta-analysis reviewed the prognostic value of lnc-SNHG1 expression in eight solid malignant tumors and indicated that the expression of lnc-SNHG1 significantly correlated with reduced OS (HR = 1.917; 95% CI, 1.58–2.31) (*P* < 0.001), TNM stage (OR = 3.99; 95% CI, 2.48–6.43), and lymph node metastasis (OR = 3.12; 95% CI, 1.95–4.98). However, no significant correlation was observed between lnc-SNHG1 expression and patient sex, tumor subtype, or tumor size [30].

This study was novel in reporting that lnc-SNHG1 was upregulated in patients with chemoresistant EOC compared with patients with chemosensitive EOC. Also, lnc-SNHG1 was mainly localized in the cytoplasm. The high expression level of lnc-SNHG1 significantly correlated with tumor stage, histological grade, nodal status, metastasis, and chemoresistance except tumor size. The univariate analysis showed that the dysregulation of lnc-SNHG1 was an independent related risk factor (*P* < 0.0001). The multivariate logistic regression analysis showed that tumor stage (*P* < 0.0001) and expression of lnc-SNHG1 (*P* = 0.01) were independent risk factors associated with the chemoresistance of serous EOC (Table 4). Kaplan–Meier analysis indicated that patients with high expression level of lnc-SNHG1 showed significantly less OS compared with patients with low expression level of lnc-SNHG1 (survival interval: 40.068 ± 5.678 vs 71.329 ± 4.730, *P* < 0.0001). DFS also significantly decreased for patients with the high expression level of lnc-SNHG1 (survival interval: 27.081 ± 5.345 vs 46.238 ± 4.022, *P* = 0.003). Xiong et al. also found that lnc-SNHG1 was upregulated in breast cancer tumors, and the high expression level of lnc-SNHG1 significantly correlated with the advanced clinical stage in breast cancer tissues [31]. Xu et al. found that lnc-SNHG1 was overexpressed in human colorectal cancer tissues, and the high expression level of lnc-SNHG1 indicated the poor survival of patients with colorectal cancer [32]. More recently, Zhang et al. reported that lnc-SNHG1 enhanced the tumorigenesis of meningioma cells through the Wnt signaling pathway by sponging miR-556-5p and thus negatively regulating the expression of miR-556-5p [33]. In this study we claimed lnc-SNHG1 acted as a sponge for miR-216b-5p and thus stimulated paclitaxel sensitivity in A2780/Taxol cells.

MiR-216b has recently been reported as a tumor suppressor miRNA in multiple tumors except ovarian cancer. Deng et al. reported that miR-216b expression was low in nasopharyngeal carcinoma cell lines and tissues; the overexpression of miR-216b could inhibit cell proliferation and invasion by targeting KRAS [34]. Xu et al. reported that miR-216b negatively regulated c-Jun and thus promoted cell apoptosis in patients with endoplasmic reticulum stress [35]. Liu et al. reported that the expression of miR-216b was significantly lower in HCC tissues than in normal tissues [36]. The overexpression of miR-216b reduced its target gene expression and thus inhibited hepatoma cell proliferation, migration, and invasion. Wang [22] et al. found that the expression of miR-216b was significantly downregulated in gastric adenocarcinoma tissues than in noncancer tissues, and the overexpression of miR-216b inhibited gastric cancer cell proliferation by negatively regulating its target gene HDAC8. However, whether the low expression level of miR-216b-5p was related to the chemoresistance of ovarian cancer was not reported at home and abroad. Applying starBase v2.0 [23], it was found that lnc-SNHG1 could directly target miR-216b-5p (Fig. S1). The expression and location of miR-216b-5p were also tested to evaluate the expression level of miR-216b-5p and further find the correlation between lnc-SNHG1 and miR-216b in patients with ovarian cancer.

The data showed that the expression level of miR-216b-5p was remarkably lower in patients with chemoresistant EOC compared with patients with chemosensitive EOC (high expression level rate: 10.81% vs 89.19%%, *P* = 0.0001); it was mainly localized in the cytoplasm. No significant association was found between miR-216b-5p expression and parameters including tumor stage, histological grade, nodal status, and metastasis. However, the low expression level of miR-216b-5p significantly correlated with chemoresistance. The univariate analysis showed that the dysregulation of miR-216b-5p was an independent risk factor for chemoresistance (*P* < 0.0001) (Table 2). Besides, the multivariate logistic regression analysis was also used. The results showed that the expression level of miR-216b-5p (*P* = 0.033) was an independent risk factor associated with the chemoresistance of patients with serous EOC (Table 3). The multivariate Cox regression analysis indicated that the dysregulation of miR-216b-5p (*P* = 0.012, RR 2.137, 95% CI 1.109–5.339) and FIGO stage (*P* = 0.001, RR 3.537, 95% CI 1.72–7.276) were independent prognostic factors for the OS of patients with serous EOC (Table 4). However, the FIGO stage (*P* = 0.003, RR 2.237, 95% CI 1.323–3.783) was the independent prognostic factor for the DFS of patients with serous EOC (Table 5). Kaplan–Meier analysis indicated that patients with the low expression level of miR-216b-5p showed shorter OS (survival interval: 51.875 ± 5.267 vs 69.373 ± 5.601, *P* = 0.010) and DFS (survival interval: 33.978 ± 4.413 vs 45.949 ± 4.823, *P* = 0.007). Spearman’s correlation analysis revealed significantly negative correlations between lnc-SNHG1 and miR-216b-5p (*r* = − 0.424, *P* = 0.0001). Interestingly, in 2018, You et al. [37] also showed that the decreased expression level of miR-216b-5p was significantly associated with large tumor size and advanced TNM stage. Moreover, the low expression level of miR-216b-5p was associated with OS by applying both Kaplan–Meier and multivariate survival analyses. The correlation between lnc-SNHG1 and miR-216b was not reported in patients with ovarian cancer.

The present study explored whether lnc-SNHG1 bound to miR-216b-5p to affect the paclitaxel sensitivity of ovarian cancer cells. Using starBase v2.0, it was found that lnc-SNHG1 could directly target miR-216b-5p (Fig. S1). The luciferase reporter assays and RIP-qPCR with the A2780/Taxol cells confirmed the direct interaction between miR-216b-5p and lnc-SNHG1. Hence, the overexpression of lnc-SNHG1 acted as a sponge for miR-216b-5p. Further, the biological functions were tested using CCK-8 assay, colony formation assay, wound healing assay, and cell apoptosis assay. The results showed that the knockdown of lnc- SNHG1 and the overexpression of miR-216b-5p stimulated paclitaxel sensitivity in A2780/Taxol cells through inhibiting cell growth and migration and promoting apoptosis. The inhibition of miR-216b-5p could rescue the effect of lnc-SNHG1 inhibition on the sensitivity of A2780/Taxol cells to paclitaxel.

In summary, this study found that the expression of Lnc-SNHG1 was upregulated while miR-216b-5p showed the low expression level in patients with chemoresistant EOC compared with patients with chemosensitive EOC. The increased expression level of lnc-SNHG1 was found to have a significant association with shorter OS and DFS; patients with the low expression level of miR-216b-5p also had shorter OS and DFS. Most importantly, the study confirmed that lnc-SNHG1 functioned as a ceRNA with miR-216b-5p, which was critical in modulating paclitaxel sensitivity of ovarian cancer cells. To our knowledge, we for the first time claimed that lnc-SNHG1 by sponging miR-216b-5p might contribute to the paclitaxel resistance in ovarian cancer cells. In our future study, we aim to find the functional target participated in its regulation.

## Methods

### Tissues and clinical data

The human ovarian cancer tissue microarray was purchased from Shanghai Outdo Biotech (Sample No. HOvaC154Su01, Shanghai, China). The tissue microarray included 2 cases of benign ovarian tumors and 152 cases of ovarian cancer followed up for 5–9 years. Of these, the serous epithelial ovarian cancer tissues were selected for this study. The selected patients were divided into two groups, namely, the chemosensitive group and the chemoresistant group, according to the NCCN guideline. The study included 63 cases of chemosensitive serous EOC tissues and 32 cases of chemoresistant serous EOC tissues. The clinical features and pathological information are summarized in Table 1. The study was approved by the ethics committee of the First Affiliated Hospital of Xi’an Jiaotong University (approval number: 2020-G143) and the ethics committee of Shanghai Outdo Biotech Company (approval number: YB M-05-02).

### RNA fluorescence in situ hybridization

The primer for the lnc-SNHG1 fluorescence in situ hybridization (FISH) probe was 5′-GCAGGAAGGGGGTGATAAAATACAGAAATG − 3′, while that for the miR-216b-5p probe was 5′- TCACATTTGCCTGCAGAGATTT-3′. The fluorescence probe of lnc-SNHG1 was labeled with Cy3 (red), and that of miR-216b-5p was labeled with FAM (green). One tissue microarray slice was hybridized with Cy3 probes specific for lnc-SNHG1, and the other slice was hybridized with FAM probes specific for miR-216b-5p. The in situ hybridization was performed as follows. (1) The slices were dewaxed to DEPC water followed by boiling in the repair solution for 15 min and cooled naturally. Then, protease K (20 μg/mL) was dripped for digestion for about 25 min. After washing with pure water, PBS was used for washing three times. (2) Prehybridization: The slices were incubated with prehybridization solution at 37 °C for 1 h. (3) Hybridization: the prehybridization solution was poured out, the hybrid solution containing the probe of lnc-SNHG1 or miR-216b-5p (concentration 6 ng/UL) was dripped, and the slices were hybridized in the incubator at 37 °C overnight. (4) Washing after hybridization: The hybridization solution was washed off with 2× sodium saline citrate (SSC) for 10 min, with 1× SSC for 5 min twice, and then with 0.5× SSC for 10 min at room temperature. (5) The slices were incubated with [4′,6-diamidino-2-phenylindole (DAPI)] dye solution in the dark for 8 min, and then anti-fluorescence quenching sealing agent was added after washing. (6) Fluorescence microscopy and image acquisition: The slices were observed and the images were collected under a Nikon positron fluorescence microscope (Nikon Eclipse Ci, Japan). The nucleus was stained blue with DAPI under UV excitation. The fluorescence using fluorescein indicated positive expression; FAM was stained green, and Cy3 was stained red.

### Cell culture

The ovarian cancer paclitaxel-resistant cell line A2780/Taxol and its parental A2780 cell line were purchased from the American Type Culture Collection. Both A2780/Taxol and A2780 cell lines were grown in RPMI 1640 medium (Hyclone, UT, USA) supplemented with 10% fetal bovine serum (Gibco Life Technologies, NY, USA) and 1% penicillin/streptomycin (Hyclone, UT, USA). Moreover, the A2780/Taxol cell line was maintained in the culture medium containing 800 ng/mL paclitaxel (Sigma–Aldrich, MO, USA). Paclitaxel was withdrawn a week before the experiment. The aforementioned cell lines were cultured at 37°C and in the presence of 5% CO_2_ and saturated humidity. The cells in the logarithmic growth phase were used for the experiment.

### Cell transfection

MiR-216-5p-mimic, miRNA mimic negative control (miRNA mimic NC), MiR-216-5p-inhibitor, miRNA inhibitor negative control (miRNA inhibitor NC), lnc-SNHG1 siRNA, and siRNA NC were chemically synthesized by GeneCreate Biotech (Wuhan, China). MiR-216-5p-mimic and miRNA mimic NC were transfected into cells at a final concentration of 100 nM using Lipofectamine 3000 (Invitrogen, CA, USA) following the manufacturer’s protocol. Lnc-SNHG1 siRNA and siRNA NC were transfected at a concentration of 100 nM. The cells after transfection were incubated in the presence of 5% CO_2_ at 37 °C for 48 h. The transfected cells were harvested for the next analysis.

### CCK-8 assay

After transfection, the cells were seeded in a 96-well plate at a density of 5 × 10^3^ cells/well. The plates were cultured in a 5% CO_2_ incubator at 37 °C. The cells were treated with paclitaxel at a concentration of 0.04 μM, 0.2 μM, 0.9 μM, 5 μM, and 23 μM for 24 h. The cell viability was assessed using the CCK-8 assay (Dojindo, Kumamoto, Japan). The absorbance of each well at the wavelength of 450 nm was read on a spectrophotometer (XFLUOR4 Version: V 4.51). At least three independent experiments were performed in quadruplicate.

### Cell apoptosis assay

After transfection, the cells were placed in a six-well plate at a density of 2 × 10^5^/2 mL. After 24 h, A2780/Taxol cells were transfected with the miR-216b-5p inhibitor (miRNA inhibitor NC) or siRNA of lnc-SNHG1–2 (siRNA NC). After 48 h of transfection, the cells were cultured in a medium containing 5 μM paclitaxel. After 24 h, the cells were collected and washed with PBS twice for apoptosis. The Annexin V-FITC cell apoptosis detection kit (BD, New Jersey, USA) was used for staining, and flow cytometry was used to detect cell apoptosis. The experiment was repeated three times.

### Wound healing assay

After transfection, the cells were placed in a six-well plate at a density of 5 × 10^5^/2 mL and cultured to 90% confluence. The wounds were generated via scratching the cell layer with 100-μL sterile plastic pipette tips. The treatment hole was gently washed with 1 mL of precooled sterile PBS three times, the drawn cells were washed out as much as possible, and images were taken under a microscope (Olympus, Tokyo, Japan) at time points of 0 h, 24 h, and 48 h.

### Clone formation ability test

The cells after transfection in different treatment groups were plated into 6-well plates at a density of 1000 cells and placed in a 5% CO_2_ incubator at 37 °C overnight. On the next day, the cells in each group were treated with 5 μM paclitaxel for 24 h. After treatment, the cells were cultured in a fresh and complete medium. They were incubated in an incubator for 2 weeks, followed by 0.1% crystal violet staining for 30 min, PBS washing three times, and statistical data cloning. The difference in colony formation was compared.

### RNA extraction, cDNA synthesis, quantitative real-time polymerase chain reaction

Total RNA was isolated using TRIzol agent (Invitrogen, CA, USA) following the protocols of the manufacturer and treated with RQ1 DNase (Promega, WI, USA) to remove DNA. Reverse transcription reactions were carried out using a ReverTra Ace quantitative polymerase chain reaction (qPCR) reverse transcription (RT) kit (Toyobo Life Science, Shanghai, China) following the manufacturer’s protocols. The expression levels of miR-216b-5p and lnc-SNHG1 were detected by qRT-PCR. The human actin gene was used as a control. The qRT-PCR was performed on a Bio-Rad S1000 with Bestar SYBR Green RT-PCR Master Mix (Toyobo). The relative expression levels of each sample were measured using the 2^−ΔΔCt^ method (Livak and Schmittgen 2001). All reactions were performed in triplicate.

### Luciferase activity assay

A2780/Taxol cells were cultured at a density of 2 × 10^4^ cells/well in 96-well culture plates and transfected with luciferase reporter constructs (400 ng), miR-216b-5p mimics (500 nM), and the internal control vector pRL-TK, pRL-SV40, or pRL-CMV (Promega, WI, USA) at a ratio of 20:1 (reporter construct:control vector) using Lipofectamine 3000 (Invitrogen) following the manufacturer’s protocol. The transfection medium was removed 5 h after transfection and replenished with a medium containing 6 μM curcumin (Sigma–Aldrich, MO, USA) solubilized in 100% dimethyl sulfoxide (DMSO) (Sigma–Aldrich, MO, USA). The luciferase activity was measured 48 h after transfection using the Dual-Luciferase Reporter Assay System (Promega, WI, USA).

### Rip

Magna RIP™RNA-Binding Protein Immunoprecipitation Kit (Millipore, Massachusetts, USA) was applied for the RIP assay which was carried out in A2780/Taxol cells. Cell lysates were mixed with magnetic beads conjugated with IgG or human anti-Ago2 antibody in RIP buffer. Finally, precipitated RNA was purified for qRT-PCR.

### Statistical analysis

SPSS version 19.0 (IBM SPSS, IL, USA) was used for statistical analysis. The data were expressed as mean ± standard deviation. The Student *t* test was used to compare quantitative variables. The chi-square test was used to determine the association of lnc-SNHG1 or miR-216b-5p with clinicopathological variables. The multivariate logistic regression analysis was applied to determine the related factors for chemoresistance. The Cox’s proportional hazard model was applied to identify the independent prognostic factors for overall survival (OS) and disease-free survival (DFS). Spearman’s correlation analysis was used to analyze the correlation between lnc-SNHG1 and miR-216b-5p expression. The OS and DFS curves were analyzed using the Kaplan–Meier test. The log-rank test was used to compare OS and DFS between chemosensitive and chemoresistant groups. A *P* value less than 0.05 indicated a statistically significant difference.

## Supplementary Information

**Additional file 1.**

## Data Availability

All data generated or analyzed in this study are included in the manuscript.

## Abbreviations

EOC: Epithelial ovarian cancer
OS: Overall survival
DFS: Disease-free survival
Lnc-SNHG1: lnc-small nucleolar RNA host gene 1
RIP: RNA Binding Protein Immunoprecipitation Assay
