# Supplementary figures and images for: Dysregulation of lnc-SNHG1 and miR-216b-5p correlate with chemoresistance and indicate poor prognosis of serous epithelial ovarian cancer
Source: J Ovarian Res. 2020 Dec 10;13:144. doi: 10.1186/s13048-020-00750-4 (PMC7731520; doi:10.1186/s13048-020-00750-4)

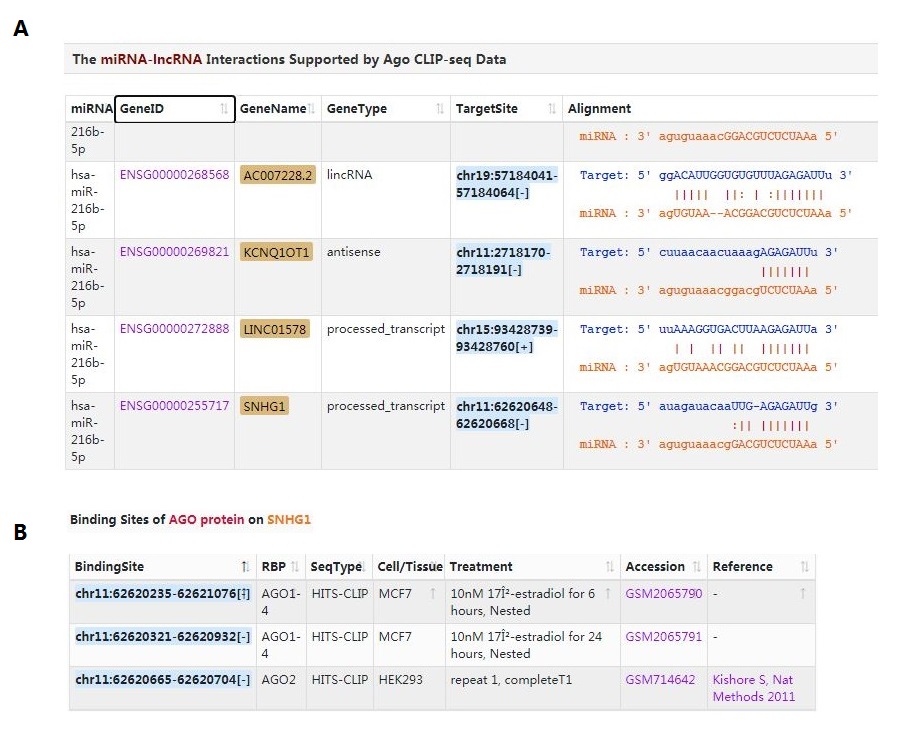

Supplement: Supplementary file 1 — Additional file 1. [file 13048_2020_750_MOESM1_ESM.jpg]
